# Seeking temporal refugia to heat stress: increasing nocturnal activity despite predation risk

**DOI:** 10.1098/rspb.2023.1587

**Published:** 2024-01-17

**Authors:** Francesca Brivio, Marco Apollonio, Pia Anderwald, Flurin Filli, Bruno Bassano, Cristiano Bertolucci, Stefano Grignolio

**Affiliations:** ^1^ Department of Veterinary Medicine, University of Sassari, via Vienna 2, Sassari 07100, Italy; ^2^ Parc Naziunal Svizzer, Zernez 7530, Switzerland; ^3^ Gran Paradiso National Park, Via Pio VII 9, Torino 10135, Italy; ^4^ Department of Life Science and Biotechnology, University of Ferrara, via Borsari 46, Ferrara, 44121, Italy

**Keywords:** activity rhythms, *Canis lupus*, *Capra ibex*, global change, moonlight, temporal shifts

## Abstract

Flexibility in activity timing may enable organisms to quickly adapt to environmental changes. Under global warming, diurnally adapted endotherms may achieve a better energy balance by shifting their activity towards cooler nocturnal hours. However, this shift may expose animals to new or increased environmental challenges (e.g. increased predation risk, reduced foraging efficiency). We analysed a large dataset of activity data from 47 ibex (*Capra ibex*) in two protected areas, characterized by varying levels of predation risk (presence versus absence of the wolf—*Canis lupus*). We found that ibex increased nocturnal activity following warmer days and during brighter nights. Despite the considerable sexual dimorphism typical of this species and the consequent different predation-risk perception, males and females demonstrated consistent responses to heat in both predator-present and predator-absent areas. This supports the hypothesis that shifting activity towards nighttime may be a common strategy adopted by diurnal endotherms in response to global warming. As nowadays different pressures are pushing mammals towards nocturnality, our findings emphasize the urgent need to integrate knowledge of temporal behavioural modifications into management and conservation planning.

## 1. Introduction

Anthropogenic impacts on natural systems have experienced a progressive acceleration during the last century. Direct influence from human activities affects more than 83% of Earth's land surface, with indirect influence reaching the remaining 17% through global processes such as pollution and climate change [1]. Organisms must adapt spatially or temporally to these changes to avoid extinction. Spatial responses, such as poleward and upward shifts, are well-documented animal responses to climate change [2]. However, migration to favourable areas can be hindered by the widespread presence of humans or the landscape structure. In such cases, animals may maintain their current distribution range and optimal thermal niche by modifying their ecological niche in the temporal dimension, i.e. their daily activity rhythms [3].

Circadian activity rhythm adjustments can be influenced by astronomical periodicities (e.g. changes in photoperiod lengths, lunar phases and seasonal recurrence) and social pressures, as well as ecological factors like food availability, environmental temperature and other meteorological conditions (*proximate* mechanism *sensu* [4]). These activity switches may also have an adaptive value (*ultimate* mechanism). The hypothesis of circadian thermoenergetics (*sensu* [4]) posits that ultimate mechanisms driving temporal switching should involve an optimization process where energy balance, survival and reproduction are weighed against each other to maximize fitness. According to this hypothesis, from a mere energetic point of view, all endothermic animals living in temperate zones are expected to benefit from a diurnal lifestyle because, during the night, temperatures are generally below the thermoneutral zone of most of these species. Therefore, they are expected to achieve a better energy balance by reducing heat loss through insulation during nocturnal resting [4]. However, increasing global warming may disrupt these expectations, as the costs of diurnal activity may rise while those of nocturnal activity may decrease [3]. In fact, in warm conditions, maximal heat dissipation capacity is believed to be a powerful driver of endotherms and therefore a deciding factor in pushing their behavioural adaptation [5]. With increasing temperatures predicted for the future, endotherms may achieve better energy balance by resting during the warmer diurnal hours and shifting their activity to the cooler nocturnal hours [3]. Nevertheless, the benefits of shifting activity should outweigh the costs for an adaptive temporal shift. For instance, for prey species, shifts to nocturnal activity can be constrained by a significant increase in predation risk [6] if their predators are more active and performant at night (e.g. felids and canids; [7]). Therefore, our study addresses two main research questions: (1) Does nocturnal activity increase with rising air temperature?; and (2) Does predation risk perception affect the shift from diurnal to nocturnal activity?

To answer these questions, we focused on large ungulates as they frequently exhibit cathemeral activity [8], are sensitive to global change [9] and have evolved from nocturnal to diurnal daily pattern as a result of carnivore avoidance [7]. As a model species, we used Alpine ibex (*Capra ibex*), a cold-adapted diurnal mountain ungulate known to be sensitive to global warming [10,11]. It is one of the most sexually dimorphic species among ungulates [12], resulting in different overheating and predation risks between the sexes. The lower surface-to-body-mass ratio and the accumulation of fat reserves during summer limits heat dissipation in males, making them more prone to overheating [13]. On the other hand, with their larger body size and longer horns compared with females, males are less affected by predation risk [14–16].

We analysed ibex nocturnal activity data from two areas characterized by different levels of predation risk, because the wolf (*Canis lupus*; i.e. the main predator of ibex) was present only in one area during the study period. We focused our analysis on the critical period from early spring to autumn, when ibex experience the highest environmental temperatures and need to acquire energy prior to the rigours of winter [10,17]. Taking into consideration some biological and environmental factors that are known to affect ungulate activity patterns, we tested the following predictions:

(1) Nocturnal activity increases during warmer days: considering heat stress avoidance as a key driver of behaviour in ibex [10], we expected an activity shift towards nocturnal activity with increasing temperature.
(2) Predation risk affects the shift to nocturnal activity: since the main predator of ibex, the wolf, is typically nocturnal [18], as recently confirmed for one of our study areas [19], we expected a reduced shift towards nighttime activity where the predator was present (prediction 2a). Additionally, since Alpine ibex have the retinal structure of diurnal animals [20], we predicted increased nocturnal activity during brighter nights when their ability to detect predators is higher (prediction 2b). Finally, considering the differential perception of predation risk in ibex of different sex–age classes [16], we expected this to affect their nocturnal activity, with lower nocturnal activity in females compared with males, particularly when a kid was present (prediction 2c).

## 2. Methods

### (a) Study areas

This study was carried out in two separate study areas located in Gran Paradiso National Park (GPNP; northwestern Italian Alps; 45° 30′ 10′′ N, 07° 18′ 36′′ Ε) and in the Swiss National Park (SNP; 46° 40′ 11′′ N, 10° 9′ 15′′ E). The altitudinal range in both study areas is similar, ranging from about 1500 to 3200/3300 m above sea level. The climatic conditions are also similar: largely continental, dry, with strong solar radiation, low air humidity and harsh winters. During the study period, the total annual precipitation ranged from 322.0 to 798.6 mm in the GPNP and from 485.8 to 957.0 mm in the SNP. The habitat composition is also similar: conifer forests, bushes (*Rhododendron* ssp.*, Vaccinium* ssp. *and Juniperus nana*), alpine grasslands (*Carex* spp.*, Festuca* spp.), steep slopes, rocks and stone ravines. However, the landscape of fear is partially different between the study areas: the main predator, the wolf, was present with a reproductive pack in GPNP (pack size ranging from 4 to 6; data from Gran Paradiso surveillance service), but not in SNP during data collection. In GPNP, previous studies have shown that wolf has a prevalent nocturnal activity [19] and ibex is included in its diet [21]. Both study areas are protected areas, but with different levels of conservation: the SNP is a strict nature reserve (IUCN Category 1a) where any human activity is prohibited, except hiking on designated footpaths. GPNP is a National Park (IUCN Category II) where hunting is strictly forbidden, but other human activities are partially or not regulated.

In the GPNP, ibex were captured by using chemical immobilization [22] between 2013 and 2017, while, in the SNP, captures were performed using box traps between 2006 and 2017. Ibex were equipped with GPS Plus collars (Vectronic Aerospace, Berlin) in both study areas. The exact year of birth was determined by counting the number of annual incremental growth rings on the horns [23]. Ages at capture ranged between 4 and 15 years for females and between 5 and 13 years for males.

This study complied with all national and regional laws dealing with ethics and animal welfare. In GPNP, ibex capture and handling protocols were approved by the Italian Ministry of Environment (protocol no. 25114/04). In SNP, all animal handling was carried out under permit from the Swiss cantonal and federal authorities (permit nos 1/2008, 2011_07, 2014_07F, 2017_12F, GR 2020_08F, GR/01/2021).

### (b) Data collection

#### (i) Activity data logging

The activity data of individual ibex (18 males and 9 females in GPNP, 12 males and 8 females in SNP; more details in electronic supplementary material, tables S1 and S2) were recorded by means of a dual-axis motion sensor (i.e. accelerometer) fitted on the collars. The accelerometer simultaneously measures along two orthogonal directions the changes in acceleration associated with the actual motion experienced by the collar. On the *x*-axis, the accelerometer was sensitive to acceleration events with forward/backward direction/axes, while on the *y*-axis, it recorded acceleration events with a sideways and rotary direction. The accelerometer had a dynamic range of ±2 g and measured activity as the change of static acceleration (gravity) and dynamic acceleration (collar) with a frequency of 4 Hz. The motion data from accelerometers, i.e. activity values, were calculated as the difference between consecutive measurements, averaged over a time interval of 4 or 5 min and given within a relative range between 0 (no difference between consecutive data) and 255 (difference of ±2 g), with the associated date and time. The activity data recorded were downloaded by means of a handheld terminal (Vectronic Aerospace, Berlin) and Yagi antenna.

#### (ii) Weather and astronomical data

Weather data such as hourly air temperature (°C) and hourly precipitation (i.e. the amount of rain expressed in millimetres of water) were provided by the Meteorological Service of Regione Autonoma Valle d'Aosta (weather station of Pont, 45° 31′ N, 7° 12′ E; 1951 m a.s.l.) and by the Federal Office for Meteorology and Climatology (weather station of Samedan, 46° 31′ N, 9° 52′ E; 1710 m a.s.l.), for the GPNP and SNP study areas, respectively. We *a priori* chose to use temperature rather than radiation (which are highly correlated) because previous research suggested that air temperature was the main driver affecting ibex spatial choices [10].

Moon illumination was calculated using the suncalc package in R [24] and was expressed as the illuminated fraction of the moon, which ranged from 0.0 (new moon) to 1.0 (full moon). Cloud cover estimates were downloaded from the NCEP/NCAR dataset [25] by using the interpolation method ‘inverse distance weighting' [26] by means of the NCEP.interp function in the RNCEP package in R [27]. Cloud cover data were expressed as the percentage of sky covered by clouds and had a spatial and temporal gridded resolution of 2.5° and 6 h, respectively. In our analyses, only cloud cover data recorded at 00.00 were used.

### (c) Data analysis

We restricted our analysis to the critical period for ibex when they experience the highest environmental temperatures and must acquire energy prior to the rigours of winter, i.e. from 1 May to 31 October [10,17,28] from 2006 to 2019.

Activity raw data were transformed into active/inactive behavioural states using a threshold value calculated for each individual during each month of data collection, following the method proposed by Brivio *et al*. [29]. Thus, we obtained a binary variable, i.e. activity state, where 0 means inactive states and 1 active states. We obtained the time of dawn and dusk for each day for each study area by using the suncalc package in R [24]. Accordingly, we classified each activity state record as diurnal or nocturnal by considering the recording date and time and the relative dawn and dusk times. After splitting the activity data into two subsets, the proportion of diurnal active time (PDAT) and the proportion of nocturnal active time (PNAT) were calculated as the average of all activity state values recorded during each day and each night, respectively. Similarly, we calculated the diurnal and nocturnal mean, maximum and minimum values for air temperature by assigning each hourly value to either a day or a night according to the recording time and then calculated the mean, maximum and minimum values for each day and each night of the data collection period. For each date of data collection, the diurnal and nocturnal precipitation values were also calculated by averaging the values recorded throughout the corresponding diurnal and nocturnal hours. Finally, for each night, we calculated night brightness according to the formula

Night brightness=moon−(cloud cover×moon),

where ‘night brightness' is the night brightness of day_i,_ ‘moon' is the illuminated fraction of the moon during the day_i_, ‘cloud cover ‘ is the cloud cover at midnight of the day_i_. Night brightness values closer to 0 represent darker nights and values closer to 1 represent brighter nights, respectively.

We then linked each PNAT to the values of temperature (mean, maximum and minimum) recorded during the corresponding night and the temperature values (mean, maximum and minimum) recorded during the previous diurnal hours. As previous research suggested that ibex might be able to sense daily temperature trends in advance [10], we also linked the temperature values (mean, maximum and minimum) recorded during the following diurnal hours. We linked mean precipitation values recorded during the night and during the previous diurnal hours. Finally, each PNAT value was linked to the PDAT values of the previous diurnal hours.

Following the data preparation described above, PNAT was modelled as the response variable by fitting alternative general additive models (GAMs) implemented within the mgcv package in R [30]. GAMs are an ideal tool for such analysis, as they are flexible in modelling the shape of nonlinear relationships. Non-parametric smoothing functions are used on sections of the data and the response curves are connected at their endpoints to generate an overall smooth curve. In addition to the non-parametric smoothing functions, parametric fixed and random predictor terms may also be included [31]. We fitted GAMs with a Tweedie distribution, which is often used when the distribution of the data includes positive continuous data points with a cluster of data at zero [32]. This data distribution is typical for activity data recorded by using accelerometers, such as data used in our analysis [33].

The predictor variables considered were the different measures of temperature, night brightness and PDAT. We included precipitation values (log-transformed in order to improve the homogeneity of the residual distribution) because they are known to affect ungulate activity patterns [22,34]. We also included the Julian date as a covariate in the models to control for the variability of ibex PNAT fluctuating throughout the monitoring period. We were not interested in the specific influence of the Julian date and precipitation on ibex nocturnal activity, but we included these variables in our models to account for their effect on activity patterns. In this way, we aimed to increase the power of our analyses to detect the effects of the variables of interest and disentangle them from other sources of variation. To investigate whether females and males living under different predation risk reacted differently to the environmental variables considered, for each variable included in the models, we added the three-way interaction with the categorical variables sex (male/female) and site (GPNP/SNP). Ibex identity was used as a random factor to control for repeated measurements of the same individual, fitting it into the GAMs by using ‘re' terms, and smoother linkage [30]. The effects of all continuous predictor variables were modelled as natural cubic spline functions. The optimal roughness of the smoothing terms was determined by minimizing the generalized cross-validation value.

Before fitting the models, possible correlations between the predictor variables were checked by means of a correlation matrix (Pearson correlation coefficient, with thresholds set to |*r*_p_| = 0.7) to avoid collinearity [35]. The nine different measures of temperatures (mean, maximum, minimum of the night; mean, maximum, minimum of the previous diurnal hours; mean, maximum, minimum of the following diurnal hours) were correlated with each other (|*r*_p_| > 0.7). Thus, we built nine alternative models, one for each measure of temperature, to test which measure best predicted the variations of PNAT. As PDAT is known to be highly affected by temperature (e.g. [3,4,36]) and our objective was to test if PDAT was a good predictor of PNAT, we also fitted an additional alternative model with PDAT as a covariate instead of temperature. Precipitation recorded during the night and precipitation recorded during the previous diurnal hours were alternatively included in the models to find the best predictor for PNAT (see electronic supplementary material, table S3 to have an overview on all the variables included in the alternative models). To check for multicollinearity between the predictor variables included in the models, we calculated the variance inflation factor (VIF). All VIF values were less than 3, indicating no severe multicollinearity between the variables [35]. The final structure of our models (*N* = 20) was:

$$\begin{array}{rl} \mathrm{PNAT}∼\; & \beta_{0}+\mathrm{sex}\times \beta_{1}+\mathrm{site}\times \beta_{2}+f_{1}(\mathrm{temperature}\times \mathrm{sex}\times \mathrm{site})\\ & \;+f_{2}(\mathrm{brightness}\times \mathrm{sex}\times \mathrm{site})\\ & \;+f_{3}(\mathrm{precipitation}\times \mathrm{sex}\times \mathrm{site})\\ & \;+f_{4}(\mathrm{date}\times \mathrm{sex}\times \mathrm{site})+(1|\mathrm{ID})+\varepsilon , \end{array}$$

where ‘sex' is a categorical variable including two levels (female/male), ‘site' is a categorical variable including two levels (area with predator/area without predator), ‘temperature' is one of the nine different measures of temperature or PDAT, ‘brightness' is night brightness, ‘precipitation' is one of the two different measures of precipitation, ‘date' is the Julian date, ‘ID’ is the identity of ibex, included as random factor.

The alternative models were ranked and weighted with the minimum Akaike information criterion (AIC; [37]; electronic supplementary material, table S4). We confirmed the global goodness-of-fit (i.e. homoscedasticity, normality of errors and independence) of the best model by visual inspection of residuals [35].

Each year, researchers and rangers from both study areas continuously monitored marked females in order to observe whether these females were followed by kids or not. Reproductive status of each female was scored using a binomial score (i.e. each individual received either a score of one when observed with a kid during summer or a score of zero if observed without a kid). We included this score in a new variable (named reproductive status) and used it to test the effect of the presence of kid on the nocturnal activity of females. We analysed a subset of our data corresponding to the periods during which we had information about female productivity (i.e. we knew if females were with or without their kid). Therefore, we followed the same approach used for the overall dataset and we fitted all alternative models, including all predictors with the three-way interaction with the categorical variables reproductive status (female with kid/female without kid) and site (GPNP/SNP; see electronic supplementary material, table S5). The final structure of our models (*N* = 20) was

$$\begin{array}{rl} \mathrm{PNAT}∼\; & \beta_{0}+\mathrm{repr}.\mathrm{status}\times \beta_{1}+\mathrm{site}\times \beta_{2}\\ & \;+f_{1}(\mathrm{temperature}\times \mathrm{repr}.\mathrm{status}\times \mathrm{site})\\ & \;+f_{2}(\mathrm{brightness}\times \mathrm{repr}.\mathrm{status}\times \mathrm{site})\\ & \;+f_{3}(\mathrm{precipitation}\times \mathrm{repr}.\mathrm{status}\times \mathrm{site})\\ & \;+f_{4}(\mathrm{date}\times \mathrm{repr}.\mathrm{status}\times \mathrm{site})+(1|\mathrm{ID})+\varepsilon , \end{array}$$

where ‘repr.status' is a categorical variable including two levels (female with kid/female without kid), ‘site' is a categorical variable including two levels (area with predator/area without predator), ‘temperature' is one of the nine different measures of temperature or PDAT, ‘brightness' is night brightness, ‘precipitation' is one of the two different measures of precipitation, ‘date' is the Julian date, ‘ID' is the identity of females, included as random factor.

## 3. Results

A total of 3 703 248 activity records (GPNP: 2 309 040, SNP: 1 394 208) were acquired during 11 255 (GPNP: 6414, SNP: 4841) monitoring days for all individuals.

Among the models fitted to explain the variation in nocturnal activity, according to the minimum AIC criterion, the model including the maximum temperature recorded during the previous diurnal hours outperformed the alternative models including other measures of temperature, as well as the model including PDAT as a predictor (electronic supplementary material, table S4). This model included interaction terms of sex and the study site (GPNP and SNP) with maximum temperature recorded during the previous diurnal hours, night brightness, precipitation recorded during the night and Julian date (*R*^2^-adj = 0.48).

Likewise, the model including maximum temperature recorded during the previous diurnal hours outperformed the alternative models fitted on the reduced dataset concerning females only, during the period for which we had information about their productivity (electronic supplementary material, table S5). The best model, selected according to the minimum AIC criterion, included the interaction terms between the reproductive status (female with kid/female without kid) and the study site with the following variables: maximum temperature recorded during the previous diurnal hours, night brightness, precipitation recorded during the night, and Julian date (*R*^2^-adj = 0.37).

**Figure 1.**
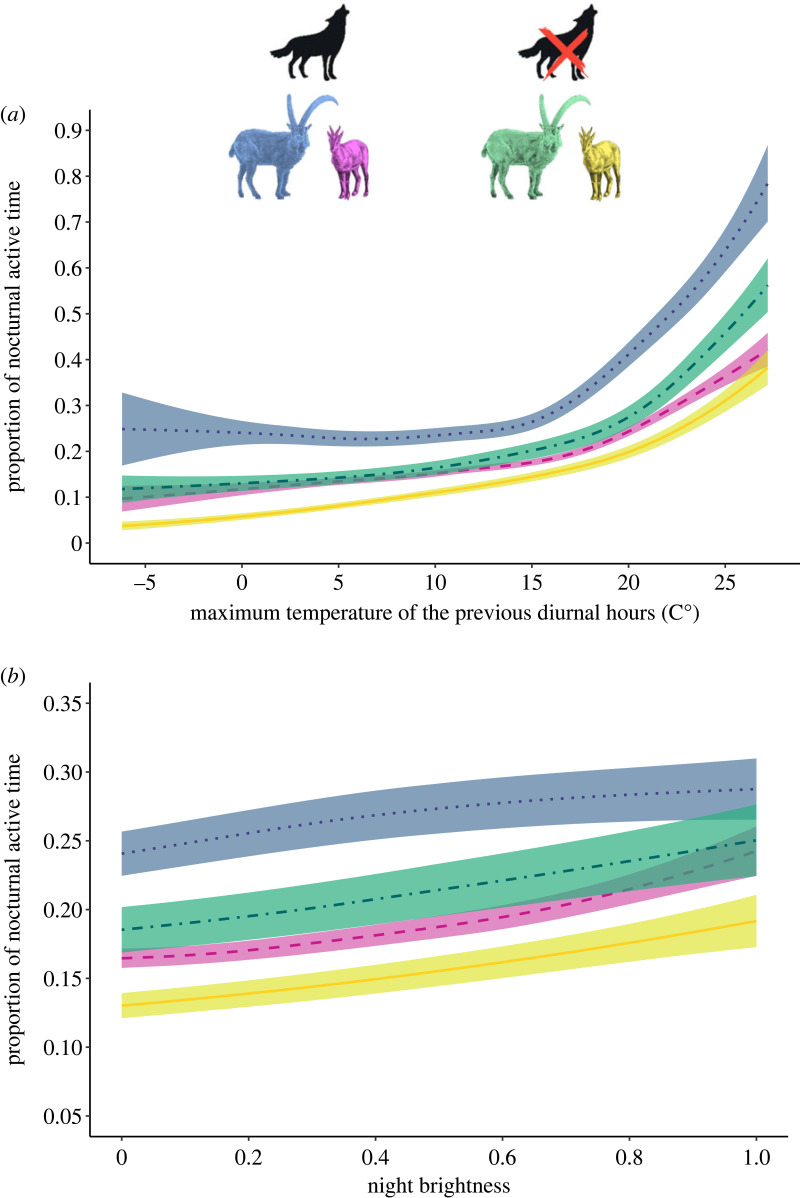
Values predicted by the best generalized additive model (see the text for more details) for the proportion of nocturnal active time of Alpine ibex (*Capra ibex*) in the Gran Paradiso National Park (GPNP, Italy) and the Swiss National Park (SNP, Switzerland). The figure shows the effects exerted by maximum temperature recorded during the previous diurnal hours (*a*) and night brightness (*b*) for males (dotted blue line) and females (dashed pink line) in the area with the predator (GPNP) and for males (dot–dashed green line) and females (solid yellow line) in the area without the predator (SNP). The predictions are given according to the mean of all other covariates in the model, for the collar ID 12507. The coloured shaded areas are the estimated standard errors.

The results of the most parsimonious model (table 1) showed that during night males were more active than females and, overall, ibex of the GPNP were more active with respect to ibex of SNP. The most important factor that affected the PNAT of ibex was the maximum temperature recorded during the previous diurnal hours. The pattern of variation of PNAT of males and females living in the area with and without the predator (GPNP and SNP, respectively) was slightly different, but the predictions of the model highlighted that the general trends were very similar: both sexes increased their nocturnal activity with increasing temperature in both the study sites (figure 1*a*). The night brightness had a positive effect on the nocturnal activity of ibex: PNAT of males and females in the two study sites increased with increasing night brightness, with only weak differences among males and females living with and without the predator (figure 1*b*). Nocturnal precipitation had a negative effect: both females and males in the two study sites decreased their activity with increasing precipitation (electronic supplementary material, figure S1a). The PNAT of both males and females increased from May to the end of October. In GPNP, females showed an intermediate peak of nocturnal activity around 27 June, while for females in SNP this peak was reached around 20 May. In males, PNAT increased throughout the monitoring period in both study areas, but in the SNP, the peak of nocturnal activity was reached around 6 October, after which their nocturnal activity appeared to decrease slightly (electronic supplementary material, figure S1b).

**Table 1.** Effect of predictor variables estimated by the best generalized additive model (see the text and electronic supplementary material, table S4 for more details) fitted to predict the proportion of nocturnal active time of Alpine ibex (*Capra ibex*) in the Gran Paradiso National Park (Italy) and in the Swiss National Park (SNP, Switzerland). In the table, 'sex' is the sex of the monitored ibex (female/male); 'site' is the study area with the predator (GPNP) or the study area without the predator (SNP); 'temp PDH max' is the maximum temperature of the previous diurnal hours; 'NB' is the night brightness; 'prec NH' is the precipitation recorded during the corresponding night (log-transformed); 'J date' is the Julian date. All the predictor variables were included in the models in interaction with sex and site: 'females GPNP' are females monitored in the area with the predator; 'males GPNP' are males monitored in the area with the predator (GPNP); 'females SNP' are females monitored in the area without the predator (SNP); 'males SNP' are males monitored in the area without the predator (SNP).

parametric coefficients:				
	estimate	s.e.	*t*-value	Pr(>|*t*|)
(intercept)	−1.503	0.050	−29.938	<0.001***
sex (males)	0.198	0.055	3.594	<0.001***
site (SNP)	−0.254	0.054	−4.728	<0.001***

**approximate significance of smooth terms:**				
	edf	Ref.df	*F*	*p*-value
s(temp PDH max) : females GPNP	4.325	9	45.887	<0.001***
s(temp PDH max) : males GPNP	5.176	9	218.653	<0.001***
s(temp PDH max) : females SNP	3.363	9	82.690	<0.001***
s(temp PDH max) : males SNP	4.500	9	90.356	<0.001***
s(NB) : females GPNP	2.280	9	8.488	<0.001***
s(NB) : males GPNP	1.865	9	5.224	<0.001***
s(NB) : females SNP	1.692	9	6.631	<0.001***
s(NB) : males SNP	1.683	9	6.071	<0.001***
s(prec NH) : females GPNP	1.767	2	123.411	<0.001***
s(prec NH) : males GPNP	1.816	2	116.250	<0.001***
s(prec NH) : females SNP	1.763	2	52.025	<0.001***
s(prec NH) : males SNP	1.341	2	35.053	<0.001***
s(J date) : females GPNP	8.163	9	79.709	<0.001***
s(J date) : males GPNP	5.229	9	179.406	<0.001***
s(J date) : females SNP	6.801	9	89.431	<0.001***
s(J date) : males SNP	5.952	9	261.194	<0.001***
s(animal ID)	41.184	44	22.601	<0.001***

**Figure 2.**
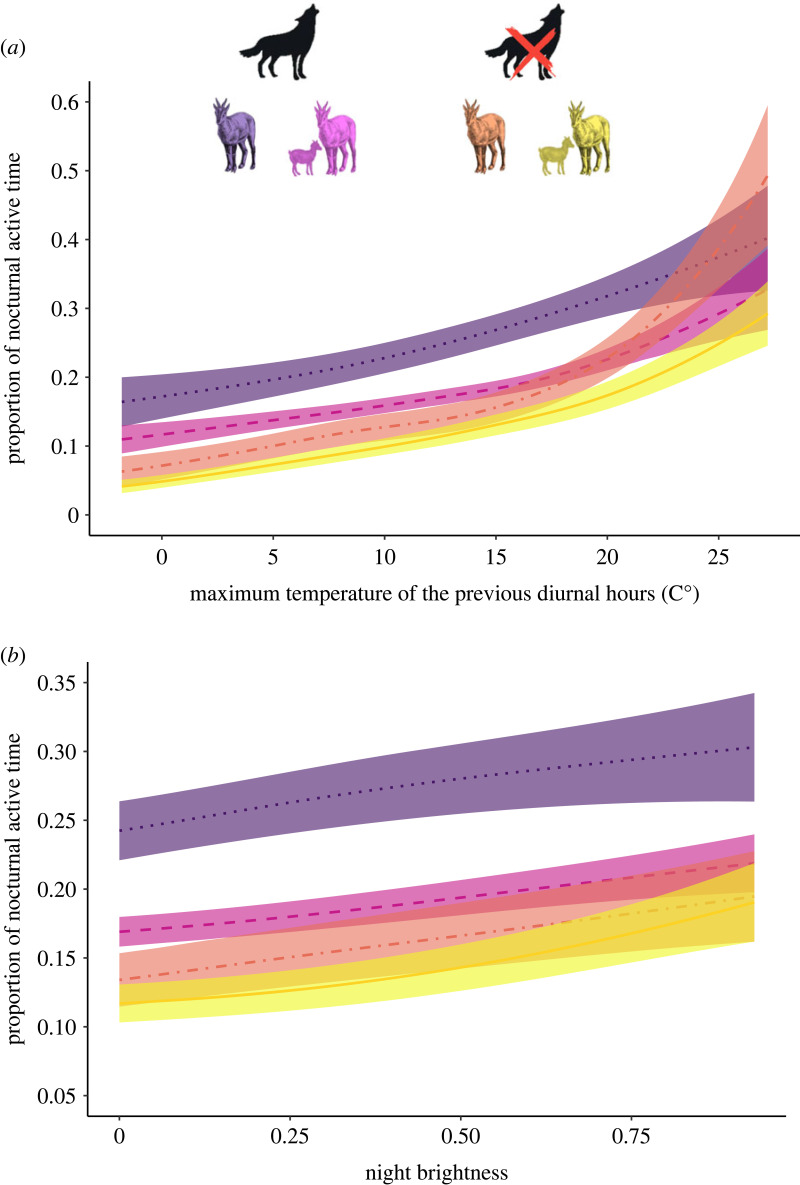
Values predicted by the best generalized additive model (see the text for more details) for the proportion of nocturnal active time of Alpine ibex (*Capra ibex*) females in the Gran Paradiso National Park (GPNP, Italy) and the Swiss National Park (SNP, Switzerland). The figure shows the effects exerted by maximum temperature recorded during the previous diurnal hours (*a*) and the night brightness (*b*) for females without kid (dotted violet line) and females with kid (dashed pink line) in the area with the predator (GPNP) and for females without kid (dot–dashed orange line) and females with kid (solid yellow line) in the area without the predator (SNP). The predictions are given according to the mean of all other covariates in the model, for the collar ID 12507. The coloured shaded areas are the estimated standard errors.

The results of the best models fitted to test the effect of the presence of kid on the PNAT of females were consistent with the results of the analyses carried out on the overall dataset (table 2). Females in the SNP were less active at night than females in GPNP and, overall, females with kid had lower PNAT with respect to females without kid. This analysis confirmed that the most important factor affecting PNAT of female ibex was the maximum temperature recorded during the previous diurnal hours. The effect of temperature was slightly different in females with and without kids in the two study sites, showing a general trend of increasing nocturnal activity with increasing temperature (figure 2*a*). We found a positive effect of night brightness on PNAT, again, with only slight differences in females with and without kids in the study sites (figure 2*b*). Precipitation recorded during the night negatively affected the nocturnal activity consistently in the two study sites and among females with and without kid (electronic supplementary material, figure S2a). The nocturnal activity of female ibex fluctuated throughout the monitoring period, with an overall pattern of increasing activity towards the end of October (electronic supplementary material, figure S2b).

**Table 2.** Effect of predictor variables estimated by the best generalized additive model (see the text and electronic supplementary material, table S5 for more details) fitted to predict the proportion of nocturnal active time of Alpine ibex (*Capra ibex*) females in the Gran Paradiso National Park (GPNP, Italy) and in the Swiss National Park (SNP, Switzerland). In the table, ‘repr st’ is the reproductive status of the monitored females (with/without kid); ‘site’ is the study area with the predator (GPNP) or the study area without the predator (SNP); ‘temp PDH max’ is the maximum temperature of the previous diurnal hours; ‘NB’ is the night brightness; ‘prec NH’ is the precipitation recorded during the corresponding night (log-transformed); ‘J date’ is the Julian date. All the predictor variables were included in the models in interaction with reproductive status and site: ‘females_kid GPNP’ are females with kid monitored in the area with the predator; ‘females_noKid GPNP’ are females without kid monitored in the area with the predator (GPNP); ‘females_kid SNP’ are females with kid monitored in the area without the predator (SNP); ‘females_noKid SNP’ are females without kid monitored in the area without the predator (SNP).

parametric coefficients:				
	estimate	s.e.	*t*-value	Pr(>|*t*|)
(intercept)	−1.405	0.070	−20.006	<0.001***
repr st (females_noKid)	−0.124	0.045	−2.747	<0.001***
site (SNP)	−0.375	0.099	−3.783	<0.001***

**approximate significance of smooth terms:**				
	edf	Ref.df	*F*	*p*-value
s(temp PDH max) : females_kid GPNP	1.549	9	2.159	<0.001***
s(temp PDH max): females_noKid GPNP	2.277	9	4.748	<0.001***
s(temp PDH max) : females_kid SNP	3.023	9	17.347	<0.001***
s(temp PDH max) : females_noKid SNP	3.179	9	22.110	<0.001***
s(NB) : females_kid GPNP	1.082	9	0.732	<0.001***
s(NB) : females_noKid GPNP	1.301	9	1.685	<0.001***
s(NB) : females_kid SNP	1.293	9	1.857	<0.001***
s(NB) : females_noKid SNP	1.762	9	4.486	<0.001***
s(prec NH) : females_kid GPNP	1.312	2	21.834	<0.001***
s(prec NH) : females_noKid GPNP	1.362	2	39.006	<0.001***
s(prec NH) : females_kid SNP	1.240	2	8.763	<0.001***
s(prec NH) : females_noKid SNP	1.215	2	19.260	<0.001***
s(J date) : females_kid GPNP	4.730	9	7.152	<0.001***
s(J date) : females_noKid GPNP	6.973	9	41.441	<0.001***
s(J date) : females_kid SNP	5.868	9	13.459	<0.001***
s(J date) : females_noKid SNP	6.326	9	34.750	<0.001***
s(animal ID)	12.154	13	18.802	<0.001***

## 4. Discussion

Our study revealed that ibex cope with warmer temperatures by becoming more nocturnal: after days with high maximum temperatures, both males and females increased their nocturnal activity, arguably to compensate for reduced diurnal food intake (prediction 1). Interestingly, we found that the primary driver of nocturnal activity was the maximum temperature recorded during the previous diurnal hours, rather than the temperature recorded during the night. Contrary to our expectation, ibex nocturnal activity was higher in the area where the nocturnal predator was present (prediction 2a). We found that females were less active at night compared with males, particularly when accompanied by a kid (prediction 2c). However, similar to males, females consistently increased their nocturnal activity in response to high diurnal temperature. When active at night, ibex appeared to benefit from moon illuminance (prediction 2b), which likely enhances sight efficiency and early predator detection. Overall, these findings suggest that, under warm conditions, nocturnal activity serves as a primary strategy for ibex to maintain their preferred thermoneutral zone, and the need to reduce thermoregulation costs likely outweighs predation risk avoidance in importance.

### (a) Nocturnal activity as a response to global warming

The increased nocturnal activity of ibex following warmer days supports the hypothesis that a shift of activity towards nighttime may be a common tactic adopted by diurnal endotherms in response to global warming. This hypothesis was experimentally tested by Levy *et al*. [3], who showed in an endothermic laboratory model that the future summer climate may decrease the costs of nocturnal activity and increase those of diurnal activity. Some researchers have also provided evidence of activity shifts as an adaptation to reduce heat stress in wild mammals living in arid environments (*Oryx leucoryx* and *Gazella subgutturosa marica*; [38–40]), and many other studies have shown that ambient temperature strongly influence animal activity budgets [36,41–44]. However, most of these studies have limited their analyses on direct responses to the actual temperature experienced by the animals. In our study, we advanced the analysis by testing different temperature measurements to understand which played a pivotal role in driving activity patterns. In so doing, we provide evidence of a delayed response in a mountain ungulate, which strongly increased its subsequent nocturnal activity above a certain threshold of the diurnal temperature. This behavioural pattern suggests a compensation strategy for reduced diurnal activity during warmer days [11,45,46]: ibex shift foraging activity to the night, likely reducing exposure to heat stress and minimizing the energetic costs associated with thermoregulation, while compensating for reduced food intake. However, since the model including the measure of the acute heat stress (i.e. maximum diurnal temperature) outperformed the model including the PDAT as a predictor, we can infer that the nocturnal shift primarily responds to overheating stress rather than directly compensating for limited diurnal food intake. These results warrant more in-depth analyses and raise new scientific questions regarding the ultimate consequences of global warming on individual fitness and population dynamics.

### (b) Increasing nocturnal activity regardless of predation risk

The shifts in nocturnal activity may be compromised by trade-offs with other factors, such as food requirements and vulnerability to predation. As food availability for herbivores is relatively constant throughout the diel cycle, food requirements are not expected to constrain their activity timing. By contrast, predator activity or hunting success can vary on a daily basis, resulting in a dynamic landscape of fear across the diel cycle [47], which may constrain the activity timing of their prey. Consequently, prey species are more likely to be active during the day when coexisting with nocturnally active predators [48]. Therefore, nocturnal carnivores may limit their prey's capacity to adapt to warmer conditions by shifting their activity to nighttime. Veldhuis *et al*. [6] provided evidence for this by comparing areas with and without lions, demonstrating that African herbivores exhibit reduced activity during cooler nocturnal hours and are more exposed to heat stress in the presence of predators. By contrast, our study revealed that ibex nocturnal activity was higher exactly in the area where the nocturnal predator (i.e. the wolf) was present. Moreover, we found consistent responses to high temperatures in both predator-present and predator-absent areas. On the one hand, these two results seem to highlight that predation risk did not affect the ibex activity rhythms. On the other hand, the lower levels of nocturnal activity of females with respect to males, particularly when a kid was present, suggest that anti-predator strategies are relevant in shaping ibex behaviour. This is in accordance with a previous study which showed that small-body females, especially when accompanied by a kid, perceived a higher predation risk than males, even in the absence of predators [16,49]. Nevertheless, we found a consistent increase in the nocturnal activity of females in response to high diurnal temperature, even when accompanied by kids and in the presence of predators. Thus, the evidence supporting the primary effect of heat stress avoidance on nocturnal activity shift (consistent responses to increased diurnal temperature by different sex–reproductive classes in both study areas, higher nocturnal activity in the area with the predator) outweighed the evidence supporting the role of predation risk (differences in nocturnal activity among sex–reproductive classes), although the latter cannot be fully neglected. In support to this conclusion, it is worth noting that body size has important consequences on the animals' ability to dissipate heat and not only on predation risk perception. Male ibex, weighing twice as much as females [14], have a lower surface–body mass ratio, which makes them more prone to overheating than females. We can conjecture that the higher nocturnal activity of males may be the consequence of their more accentuated heat stress avoidance and not just a consequence of their lower predation risk perception. Therefore, we may conclude that, overall, the findings of this study suggest that the allocation of energy by endothermic organisms is primarily governed by their ability to avoid heat stress, rather than their need to avoid predation. This confirmed the Heat Dissipation Limit theory [5], which gives an overwhelming importance to the capacity of endotherm to dissipate heat with respect to other environmental requirements.

### (c) Taking advantage of moon illuminance

We found that ibex slightly increased nocturnal activity with increasing night brightness. These findings confirmed our expectations (prediction 2b) and suggested that ibex took advantage of moon illuminance to enhance their nocturnal activity. The visual system is a key feature of the evolutionary adaptation of animals to specific temporal niches [7], and it was thought to be one of the main constraints on activity shifts [4]. For diurnal species relying on vision, low light levels at night can reduce foraging success, movement efficiency and early detection of predators. Although it should be considered that nocturnal movements in risky environments, such as steep terrains and rocky slopes, can be safer and more efficient during brighter nights, our results pointed out that the nocturnal activity of ibex was more pronounced in the area where the predator was present, suggesting a role of predation risk in this choice. Most ungulate species exhibit higher nocturnal activity during brighter nights [50], despite moonlight also improving the hunting success of most of their predators [18,51–54]. It is worth noting that in the specific case of ibex–wolf system the former is a diurnal mammal whereas the latter is a nocturnal one, so the benefit of a bright night is comparatively higher for the prey than for the predator. Therefore, we can conclude that, for most ungulates relying on vision as their primary sensory system, the benefits of being active during brighter nights (improved food and predator detection, safer movements) outweigh the costs (increased vulnerability to predation; [55]).

## 5. Conclusion

Behavioural adaptations represent the fastest responses of animals to environmental stressors, often outpacing evolutionary adaptations and incurring lower costs compared with physiological responses [56]. Therefore, behavioural adaptations, such as activity time flexibility, are expected to be prominent in response to rapid changes and may provide organisms with a rapid pathway to accommodate environmental variations. The observed activity shift towards nocturnal hours indicates that, on the whole, large mammals such as ibex may be able to mitigate the effects of global warming by seeking refuge during cooler nocturnal hours [3]. Nowadays, various pressures are pushing mammals towards nocturnality. Increasing scientific research suggests that human disturbances—ranging from urban development and agriculture to lethal (e.g. hunting and persecution) and nonlethal activities (e.g. hiking and natural resource extraction)—are driving a global increase in nocturnal activity across numerous mammal species [57]. If, on the one hand, this shift towards nocturnality may facilitate animal adaptations to anthropogenic global change, on the other hand, it may bring negative consequences at individual, population and community levels. When active at night, diurnally adapted mammals may suffer from reduced foraging efficiency, weakened antipredator behaviours, restricted movement capacity, and ultimately reduced reproduction and survival rates. In fact, although most mammals have some physiological and morphological adaptations to nighttime activity owing to their nocturnal mammalian ancestors, many species have evolved traits optimized for diurnal activity [58,59]. Consequently, a mismatch between animal adaptations and environmental conditions can be expected owing to the nocturnal activity shift. Moreover, changes in activity timing can disrupt interspecific interactions such as competition and parasitism [60]. In this framework, cathemeral species are likely to be favoured as they retain the ability to adapt their activity rhythms to changing environments or better exploit resources [8,61]. Researchers and managers should pay particular attention to strictly diurnal species, as they are expected to suffer the most important impacts. Lastly, the cumulative effects of global warming and human disturbances on animal behaviour timing may negatively impact the outcomes of management activities. For example, shifts to nocturnal activity could severely hinder our ability to detect animals during the day, leading to obvious implications for the outcomes of population estimates derived from censuses. Overall, there is an urgent need to incorporate knowledge of temporal dynamics into management and conservation planning.

## Data Availability

The datasets generated and analysed during the current study are available in the Dryad Digital Repository: https://doi.org/10.5061/dryad.1ns1rn91s [62]. Supplementary material is available online [63].
